# Infrageneric Phylogeny and Temporal Divergence of *Sorghum* (Andropogoneae, Poaceae) Based on Low-Copy Nuclear and Plastid Sequences

**DOI:** 10.1371/journal.pone.0104933

**Published:** 2014-08-14

**Authors:** Qing Liu, Huan Liu, Jun Wen, Paul M. Peterson

**Affiliations:** 1 Key Laboratory of Plant Resources Conservation and Sustainable Utilization, South China Botanical Garden, Chinese Academy of Sciences, Guangzhou, China; 2 University of Chinese Academy of Sciences, Beijing, China; 3 Department of Botany, National Museum of Natural History, Smithsonian Institution, Washington, D.C., United States of America; National Institute of Plant Genome Research, India

## Abstract

The infrageneric phylogeny and temporal divergence of *Sorghum* were explored in the present study. Sequence data of two low-copy nuclear (LCN) genes, phosphoenolpyruvate carboxylase 4 (*Pepc4*) and granule-bound starch synthase I (*GBSSI*), from 79 accessions of *Sorghum* plus *Cleistachne sorghoides* together with those from outgroups were used for maximum likelihood (ML) and Bayesian inference (BI) analyses. Bayesian dating based on three plastid DNA markers (*ndhA* intron, *rpl32-trnL*, and *rps16* intron) was used to estimate the ages of major diversification events in *Sorghum*. The monophyly of *Sorghum* plus *Cleistachne sorghoides* (with the latter nested within *Sorghum*) was strongly supported by the *Pepc4* data using BI analysis, and the monophyly of *Sorghum* was strongly supported by *GBSSI* data using both ML and BI analyses. *Sorghum* was divided into three clades in the *Pepc4*, *GBSSI*, and plastid phylograms: the subg. *Sorghum* lineage; the subg. *Parasorghum* and *Stiposorghum* lineage; and the subg. *Chaetosorghum* and *Heterosorghum* lineage. Two LCN homoeologous loci of *Cleistachne sorghoides* were first discovered in the same accession. *Sorghum arundinaceum*, *S. bicolor*, *S.* x *drummondii*, *S. propinquum*, and *S. virgatum* were closely related to *S.* x *almum* in the *Pepc4*, *GBSSI*, and plastid phylograms, suggesting that they may be potential genome donors to *S. almum*. Multiple LCN and plastid allelic variants have been identified in *S. halepense* of subg. *Sorghum*. The crown ages of *Sorghum* plus *Cleistachne sorghoides* and subg. *Sorghum* are estimated to be 12.7 million years ago (Mya) and 8.6 Mya, respectively. Molecular results support the recognition of three distinct subgenera in *Sorghum*: subg. *Chaetosorghum* with two sections, each with a single species, subg. *Parasorghum* with 17 species, and subg. *Sorghum* with nine species and we also provide a new nomenclatural combination, *Sorghum sorghoides*.

## Introduction

Cultivated sorghum [*Sorghum bicolor* (L.) Moench] ranks fifth in both production and planted area of cereal crops worldwide, only behind wheat, rice, maize, and barley [1]. *Sorghum* Moench comprises 31 species exhibiting considerable morphological and ecological diversity [2]–[4] in global tropical, subtropical, and warm temperate regions [5]. The genus has panicles bearing short and dense racemes of paired spikelets (one sessile, the other pedicelled), whose sessile spikelets resemble the single sessile spikelets of *Cleistachne* Benth. These two genera were assigned to Sorghinae Clayton & Renvoize [6], one of the 11 subtribes of the tribe Andropogoneae Dumort. [7]. Previous studies of the genus using chloroplast DNA (cpDNA) and nuclear ribosomal DNA (nrDNA) internal transcribed spacer (ITS) sequences indicated that *Cleistachne* was sister to or part of an unresolved polytomy within *Sorghum* [8]–[10]. The ambiguous relationship between *Sorghum* and *Cleistachne* is reflected by the absence of pedicelled spikelets and the unverified hypothesis for the allotetraploid origin of *Cleistachne sorghoides* Benth. [2], [11]. Within Andropogoneae, *Sorghastrum* Nash has sometimes been considered as a subgenus in *Sorghum* due to its somatic chromosome number of 40 [2], or a distinct genus whose pedicelled spikelets are reduced to vestigial pedicels [12]. Therefore, the generic limits of *Sorghum* have long been a controversial issue that needs to be tested using highly informative molecular markers.

Five morphological subgenera are recognized in *Sorghum*: *Sorghum*, *Parasorghum*, *Stiposorghum*, *Chaetosorghum*, and *Heterosorghum* [2], [3], [8]. Subgenus *Sorghum* contains ten species (including the cultivated sorghum) that are distributed throughout Africa, Asia, Europe, Australia, and the Americas [2], [5]. The seven species of subg. *Parasorghum* occur in Africa, Asia, and northern Australia, and the ten species of subg. *Stiposorghum* occur in northern Australia and Asia. Subgenera *Chaetosorghum* and *Heterosorghum* are native to northern Australia and the Pacific Islands [3]. Culm nodes are glabrous or slightly pubescent in three subgenera: *Sorghum*, *Chaetosorghum*, and *Heterosorghum*, and bear a ring of hairs in subg. *Parasorghum* and *Stiposorghum* [2], [13]. Subgenus *Sorghum* is characterized by the presence of well-developed pedicelled spikelets, while subg. *Chaetosorghum* and *Heterosorghum* are characterized by pedicelled spikelets which are reduced to glumes [2], [3].

The five morphological subgenera of *Sorghum* are not shown to be concordant with molecular phylogenetic hypothesis [14]–[16]. The combined ITS1/*ndhF*/*Adh1* sequence data support a clade of *Sorghum* plus *Cleistachne sorghoides* that is divided into two lineages, one containing subg. S*orghum*, *Chaetosorghum* and *Heterosorghum*, as well as *Cleistachne sorghoides*, and the other, subg. *Parasorghum* and *Stiposorghum* [14]. Uncertainty about relationships in *Sorghum* has led to the reclassification of three distinct genera: *Sarga* Ewart including species of subg. *Parasorghum* and *Stiposorghum*; *Sorghum* including *S. bicolor*, *S. halepense* (L.) Pers., and *S. nitidum* (Vahl) Pers.; and *Vacoparis* Spangler including species of sub. *Chaetosorghum* and *Heterosorghum* [15]. Ng’uni et al. [16] argued that this reclassification was unwarranted. Based on plastid and ITS sequence data, they found that *Sorghum* consisted of two lineages: one lineage containing species of subg. *Sorghum*, *Chaetosorghum* and *Heterosorghum*, and a second lineage containing species of subg. *Parasorghum* and *Stiposorghum*. More than 80% of samples were confined to Australia in previous molecular studies, which focused on resolving interspecific relationships in subg. *Sorghum*. Therefore, the molecular analysis based on a greater sampling of taxa throughout their geographic ranges is essential to explore the infrageneric relationships in *Sorghum*.

The species of *Sorghum* are an excellent group for understanding the evolutionary patterns in crop species and wild relatives since the genus contains a large tertiary gene pool (GP-3, a genetic entity developed by Harlan and De Wet [17] to deal with varying levels of interfertility among related taxa), and a relatively small secondary gene pool (GP-2) [9]. Members of primary gene pool (GP-1) from the same species (such as the cereal species) can interbreed freely. Members of GP-2 are closely related to members of GP-1, although there are some hybridization barriers between members of GP-1 and GP-2, which can occasionally produce fertile first-generation (F1) hybrids. Members of GP-3 are more distantly related to members of GP-1, while gene transfers between members of GP-1 and GP-3 are impossible without artificial disturbance measures [17]. Members of subg. *Sorghum* are found in GP-2, except for *S. bicolor*, which belongs to GP-1, while species of the other four subgenera are found in GP-3 [18]. Subgenus *Sorghum* is traditionally treated as two complexes: the *Arundinacea* complex, consisting of annual non-rhizomatous species such as *S. arundinaceum* (Desv.) Stapf, *S. bicolor*, *S.* x *drummondii* (Nees ex Steud.) Millsp. & Chase, and *S. virgatum* (Hack.) Stapf; and the *Halepensia* complex, consisting of perennial rhizomatous species such as *S. almum* Parodi, *S. halepense* (L.) Pers, *S. miliaceum* (Roxb.) Snowden, and *S. propinquum* (Kunth) Hitchc. [19]. Members of GP-3 contain wild genetic resources of important agronomic traits, e.g., drought tolerance and disease resistance. Nevertheless, the studies of interspecific relationships among GP-3 species has lagged behind due to small sampling, so a detailed understanding of relationships among GP-3 species is conducive for the exploitation of these valuable agronomic traits.

To date, 21.8% of grass species have been documented to have arisen as a result of hybridization events [20], [21]. Plastid genes are commonly employed in phylogenetic reconstructions because they exist in high copy numbers in plant genomes and sequencing them often does not require cloning steps, and they are uniparentally (in most cases, maternally) inherited in angiosperms [22]. Low-copy nuclear (LCN) genes harbor the genetic information of bi-parental inheritance and often provide critical phylogenetic information for tracking evolution of plant lineages involving hybridization and allopolyploidization [23], [24]. For these reasons, LCN gene data complementing plastid gene data are more effective in identifying allopolyploids and their genome donors. Several studies using this method have successfully resolved the backbone phylogenetic patterns of economically important crop genera, e.g., *Eleusine* Gaertn. [25], *Gossypium* L. [26], and *Hordeum* L. [27].

The middle Miocene-Pliocene interval of 1.8–17.6 million years ago (Mya) was a crucial period in the diversification of Poaceae [28]. The C_4_ clades within the subfamily Panicoideae originated in the middle Miocene (ca. 14.0 Mya) in global tropical and subtropical regions. Subsequently, the ecological expansion of C_4_ Panicoideae became associated with climate aridification and cooling through the late Miocene-Pliocene boundary (3.0–8.0 Mya) [29], [30]. *Sorghum*, documented as an ecologically dominant member during the C_4_ grassland expansion [28], is characterized by its modern geographic distribution spanning five continents [5], [6], [31]. Therefore, its ecological abundance in the late Tertiary, coupled with its wide geographic distribution in modern times, implies that *Sorghum* may have established conservative ecological traits during the early diversification process, i.e., *Sorghum* is a niche-conservative C_4_ genus [32], [33]. However, the paucity of accurate age estimations of major diversification events in *Sorghum* has impeded our understanding of whether temporal relationships existed between the diversification of *Sorghum* and palaeoclimatic fluctuations during the middle Miocene-Pliocene interval. Our study will shed some light on the impact of palaeoclimatic fluctuations on the diversification of niche-conservative C_4_ grasses.

Here we explore the infrageneric phylogeny and temporal divergence of *Sorghum* by employing sequence data from two LCN and three plastid genes. The study aims to: (1) reconstruct infrageneric phylogenetic relationships in *Sorghum*; (2) investigate interspecific phylogenetic relationships among GP-3 species; and (3) estimate divergence times of major lineages in order to understand the impact of palaeoclimatic fluctuations on the diversification of *Sorghum*.

## Materials and Methods

### Plant Sampling and Sequencing

We sampled 79 accessions of 28 species in *Sorghum* [34]–[40], covering the morphological diversity and the geographic ranges of five subgenera (Table 1), plus the monotypic genus *Cleistachne*, together with seven species in six allied genera as outgroups [41], [42]. Seeds were obtained from International Livestock Research Institute (ILRI), International Crops Research Institute for the Semi-Arid Tropics (IS), and United States Department of Agriculture (USDA). Leaf material was obtained from seedlings and dry herbarium specimens deposited at CANB, IBSC, K, and US (Table S1 [2], [43]–[46]).

**Table 1:** Species of *Sorghum* included in the study. Chromosome numbers are based on the literature review.

Subgenus	Species	Longevity	Distribution	2n	References forChromosome number
*Sorghum*					
	*S. almum* Parodi	Perennial	Americas, Australia, Asia	40	[34], [35]
	*S. arundinaceum* (Desv.) Stapf	Annual	Africa, Asia, Australia, America	20	[11]
	*S. bicolor* (L.) Moench	Annual	Africa, Europe, Asia,	20	[16], [36]
			Australia, America		
	*S.* x *drummondii* (Nees ex Steud.)Millsp. & Chase	Annual	Africa, Asia, Australia, America	20	[11]
	*S. halepense* (L.) Pers.	Perennial	Mediterranean, Africa,	40	[16], [37]
			Asia, Australia		
	*S. miliaceum* (Roxb.) Snowden	Perennial	Asia, Africa, Australia	20	[38]
	*S. propinquum* (Kunth) Hitchc.	Perennial	Asia	20	[16]
	*S. sudanense* (Piper) Stapf	Annual	Africa, Asia, America, Europe	20	[39]
	*S. virgatum* (Hack.) Stapf	Annual	Africa, Asia	20	[34], [40]
*Parasorghum*					
	*S. grande* Lazarides	Perennial	Australia	30/40	[3]
	*S. leiocladum* (Hack.) C.E. Hubb.	Perennial	Australia	20	[2], [3], [16]
	*S. matarankense* E.D. Garber & L.A. Snyder	Annual	Australia	10	[3], [16]
	*S. nitidum* (Vahl) Pers.	Perennial	Asia, Australia	20/rarely 10	[2], [3], [16]
	*S. purpureosericeum* (Hochst. ex A. Rich.)	Annual	Africa, Asia	10	[2], [16]
	Asch. & Schweinf.				
	*S. timorense* (Kunth) Büse	Annual	Australia	10/rarely 20	[2], [3], [16]
	*S. versicolor* Andersson	Annual	Africa, Asia	10	[16]
*Stiposorghum*					
	*S. amplum* Lazarides	Annual	Africa, Australia	10/30	[3], [36]
	*S. angustum* S.T. Blake	Annual	Australia	10	[3], [16], [36]
	*S. brachypodum* Lazarides	Annual	Australia	10	[3], [16]
	*S. bulbosum* Lazarides	Annual	Australia	10	[3]
	*S. ecarinatum* Lazarides	Annual	Australia	10	[3], [16]
	*S. exstans* Lazarides	Annual	Australia	10	[3], [16]
	*S. interjectum* Lazarides	Perennial	Australia	30	[3], [16]
	*S. intrans* F. Muell. ex Benth.	Annual	Australia	10	[2], [3], [16]
	*S. plumosum* (R.Br.) P. Beauv.	Perennial	Asia, Australia	10/20/30	[2], [3]
	*S. stipoideum* (Ewart & Jean White)C.A. Gardner & C.E. Hubb.	Annual	Australia	10	[3], [16]
*Chaetosorghum*					
	*S. macrospermum* E.D. Garber	Annual	Australia	40	[2], [3]
*Heterosorghum*					
	*S. laxiflorum* F.M. Bailey	Annual	Australia, Asia	40	[2], [3]

Two LCN genes, phosphoenolpyruvate carboxylase 4 (*Pepc4*) and granule-bound starch synthase I (*GBSSI*), were chosen for this study. The housekeeping *Pepc4* gene encodes PEPC enzyme responsible for the preliminary carbon assimilation in C_4_ photosynthesis [47], whereas *GBSSI* gene encodes *GBSSI* enzyme for amylose synthesis in plants and prokaryotes [48]. These two LCN genes have been used for accurate phylogenetic assessments in Poaceae [49], [50]. They are predominantly low-copy in Poaceae, making it possible to establish orthology and track homoeologues arising by allopolyploidy [25], [51]. Based on genome-wide researches on cereal crops, these two LCN genes appear to be on different chromosomes [48], [52], thus each of the LCN markers can provide an independent phylogenetic estimation.

Genomic DNA extraction by means of DNeasy Plant Mini Kit (Qiagen, Valencia, CA, USA) was undertaken in accordance with the manufacturer’s instructions. Two LCN markers were amplified using primers and protocols listed in Table 2 [53], [54]. PCR products were purified by the PEG method [55]. Cycle sequencing reactions were conducted in 10 µL volumes containing 0.25 µL of BigDye v.3.1, 0.5 µL of primer, 1.75 µL of sequencing buffer (5×) and 1.0 µL of purified PCR product. For accessions that failed direct sequencing, the purified PCR products were cloned into pCR4-TOPO vectors and transformed into *Escherichia coli* TOP10 competent cells following the protocol of TOPO TA Cloning Kit (Invitrogen, Carlsbad, CA, USA). Transformed cells were plated and grown for 16 h on LB agar with X-Gal (Promega, Madison, WI, USA) and ampicillin (Sigma, St. Louis, MO, USA). We started with fewer colonies and picked more to ensure results, and eight to 24 colonies were selected from each individual via blue-white screening in order to assess allelic sequences and PCR errors [56], [57]. Inserts were sequenced with primers T7 and T3 on the ABI PRISM 3730XL DNA Analyzer (Applied Biosystems, Forster City, CA, USA).

**Table 2:** Primer sequences and PCR protocols in the study.

Region	Location	Primers	Sequence (5′–3′)	PCR parameters	Reference
***Pepc4***	Chromosome 10	*Pepc4-*8F	GAT CGA CGC CAT CAC CAC	95°C/3 min; 16×(94°C/20 s; 65°C/40s, −1°C/cycle; 72°C/90 s), 21×(94°C/20s; 50°C/40 s; 72°C/90 s); 72°C/5 min	This study
		*Pepc4-*10R	GGA AGT TCT TGA TGT CCTTGT CG		This study
***GBSSI***	Chromosome 7	*waxy*-8F	ATC GTC AAC GGC ATG GACGT	95°C/3 min; 16×(94°C/20 s; 65°C/40s, −1°C/cycle; 72°C/90 s), 21×(94°C/20s; 50°C/40 s; 72°C/90 s); 72°C/5 min	This study
		*waxy*-13R	GTT CTC CCA GTT CTT GGCAGG		This study
***ndhA*** ** intron**	Plastid	*ndhA* intron-1F	GCT GAC GCC AAA GAT TCCAC	95°C/3 min; 37×(94°C/40 sec; 51°C/40S; 72°C/100 sec); 72°C/10 min	This study
		*ndhA* intron-1R	GTA CTA GCA ATA TCT CTACG		This study
***rpl32-trnL***	Plastid	*rpl32*-F	CAGT TCC AAA AAA ACG TACTTC	The same as above	[53]
		*rpl32-trnL* ^(UAG)^	CTG CTT CCT AAG AGC AGCGT		[53]
***rps16*** ** intron**	Plastid	*rps16*-F2	AAA CGA TGT GGT AGA AAGCAA C	The same as above	[54]
		*rps16*-R2	ACA TCA ATT GCA ACG ATTCGA TA		[54]

Cloned sequences of nuclear loci were initially aligned with MUSCLE v.3.8.31 [58] and adjusted in Se-Al v.2.0a11 (http://tree.bio.ed.ac.uk/software/seal/). Subsequently, the corrected clones were assembled into individual-specific alignments that were analyzed separately using a maximum parsimony optimality criterion with the default parsimony settings in PAUP* v.4.0b10 [59]. The resulting trees were used to determine unique alleles present in each individual [56]. Alleles were recognized when one or more clones from a given individual were united by one or more characters [60]. After identifying all sequence clones for a given allele, the sequences were combined in a single project in Sequencher v.5.2.3 (Gene Codes Corp., Ann Arbor, Michigan, USA) and manually edited using a “majority-rule” criterion to form a final consensus allele sequence, and instances of PCR errors [56], [57] were easily identified and never occurred in more than one sequence. Newly obtained consensus sequences of 62 *Pepc4* alleles and 76 *GBSSI* alleles were submitted to GenBank (http://ncbi.nlm.nih.gov/genbank; Table S1).

Three plastid markers (*ndhA* intron, *rpl32-trnL*, and *rps16* intron) were amplified and sequenced to estimate lineage ages in *Sorghum*. Primer sequences and amplification protocols for the plastid markers were listed in Table 2. PCR products were purified by the PEG method [55]. Cycle sequencing reactions were conducted in 10 µL volume and were run on an ABI PRISM 3730XL DNA Analyzer. Both strands were assembled in Sequencher v.5.2.3. Sequence alignment was initially performed using MUSCLE v.3.8.31 [58] in the multiple alignment routine followed by manual adjustment in Se-Al v.2.0a11. The *Pepc4*, *GBSSI*, and combined plastid matrices were submitted to TreeBASE (http://purl.org/phylo/treebase/phylows/study/TB2:S15625).

### Phylogenetic analyses

Each data set was analyzed with maximum likelihood (ML) using GARLI v.0.96 [61], and Bayesian inference (BI) using MrBayes v.3.2.1 [62]. The substitution model for different data partitions was determined by the Akaike Information Criterion (AIC) implemented in Modeltest v.3.7 [63], and the best-fit model for each data set was listed in Table 3. ML topology was estimated using the best-fit model, and ML bootstrap support (MLBS) of internal nodes was determined by 1000 bootstrap replicates in GARLI v.0.96 with runs set for an unlimited number of generations, and automatic termination following 10,000 generations without a significant topology change (lnL increase of 0.01). The output file containing the best trees for bootstrap reweighted data was then read into PAUP* v.4.0b10 [59] where the majority-rule consensus tree was constructed to calculate bootstrap support values.

**Table 3:** Sequence and tree statistics for LCN and plastid genes used in this study.

Region	N	Averagesequencelength	Alignedsequencelength (SL)	GC%	VC	PIC	PIC/SL	Ti/Tv	Model
*Pepc4*	62	1056	1225	62.81%	415	249	20.3%	0.7428	GTR+I+G
*GBSSI*	76	1130	1501	54.66%	899	658	43.8%	0.7819	TIM+G
*ndhA* intron	62	1041	1131	33.30%	123	36	3.2%	1.1150	TVM+G
*rpl32-trnL*	62	730	807	28.45%	106	48	5.9%	0.6217	HKY+G
*rps16* intron	62	872	920	34.26%	98	29	3.2%	0.4432	F81+I+G
Combined plastid	62	2223	2858	32.28%	327	113	4.0%	0.6870	TVM+I+G

GC = guanine and cytosine; N = number of sequences; VC = variable characters; PIC = parsimony informative characters; Ti/Tv = transition/transversion ratio.

Bayesian inference (BI) analyses were conducted in MrBayes v.3.2.1 [62] using the best-fit model for *Pepc4* and *GBSSI* loci (Table 3). Each analysis consisted of two independent runs for 40 million generations; trees were sampled every 1000 generations, and the first 25% were discarded as burn-in. The majority-rule (50%) consensus trees were constructed after conservative exclusion of the first 10 million generations from each run as the burn-in, and the pooled trees (c. 60,000) were used to calculate the Bayesian posterior probabilities (PP) for internal nodes using the “sumt” command. The AWTY (Are We There Yet?) approach was used to explore the convergence of paired MCMC runs in BI analysis [64]. The stationarity of two runs was inspected by cumulative plots displaying the posterior probabilities of splits at selected increments over an MCMC run, and the convergence was visualized by comparative plots displaying posterior probabilities of all splits for paired MCMC runs.

The nuclear data were used to help determine bi-parental contributions, and multiple alleles were present for most polyploid taxa. Thus, the nuclear data cannot be combined with the plastid dataset, which provided the maternal phylogenetic framework. We rooted the *Pepc4* tree using species of *Apluda*, *Bothriochloa*, *Chrysopogon*, *Dichanthium* and *Sorghastrum* as outgroups and rooted the *GBSSI* tree using species of *Bothriochloa*, *Dichanthium*, *Microstegium* and *Sorghastrum* as outgroups [41], [42] because clean *GBSSI* sequences of *Apluda* and *Chrysopogon* could not be isolated in the laboratory. The appropriate choice of outgroups was confirmed by phylogenetic proximity (the monophyletic ingroup being supported), genetic proximity (short branch length being observed) and base compositional similarity (ingroup-like GC%; Table 3) [65].

### Molecular Dating

For molecular dating analyses using the plastid markers, a strict molecular clock model was rejected at a significance level of 0.05 (IL = 686.7024, d.f. = 60, *P* = 0.025) based on a likelihood ratio test [66]. A Bayesian relaxed clock model was implemented in BEAST v.1.7.4 [67] to estimate lineage ages in *Sorghum*. Three plastid markers were partitioned using BEAUti v.1.7.4 (within BEAST) with the best-fit model determined by Modeltest v.3.7 (Table 3).

The Andropogoneae crown age was estimated at 17.1±4.1 Mya [49] and within this confidence interval [68], although the most reliable fossils of subfamily Panicoideae were the petrified vegetative parts from the Richardo Formation in California [69] now dated to be approximately 12.5 Mya [70]–[72]. Because the lineages may have occurred earlier than the fossil record [73], the *Sorghum* stem age was set as a normal prior distribution (mean 17.1, SD 4.1). A Yule prior (Speciation: Yule Process) was employed. An uncorrelated lognormal distributed relaxed clock model was used, which permitted evolutionary rates to vary along branches according to lognormal distribution. Following optimal operator adjustment, as suggested by output diagnostics from preliminary BEAST runs, two independent MCMC runs were performed with 40 million generations, each run sampling every 1000 generations with the 25% of the samples discarded as burn-in. All parameters had a potential scale reduction factor [74] that was close to one, indicating that the posterior distribution had been adequately sampled. The convergence between two runs was checked using the “cumulative” and “compare” functions implemented in the AWTY [64]. A 50% majority rule consensus from the retained posterior trees (c. 60,000) of three runs were obtained using TreeAnnotator v.1.7.4 (within BEAST) with a PP limit of 0.5 and mean lineage heights.

## Results

### Phylogenetic analyses of *Pepc4* sequences

The aligned *Pepc4* matrix comprised 1225 characters, including partial exons 8 and 9, complete intron 9, at lengths of 841 bp, 190 bp, and 194 bp, respectively (Table 3). The *Pepc4* data provided a relatively high proportion of parsimony-informative characters (249 bp; 20.3%). The log likelihood scores of 56 substitution models ranged from 5883.8525 to 6165.2119, and Modeltest indicated that the best-fit model under AIC was GTR+I+G with base frequencies (π_A_ = 0.19, π_C_ = 0.32, π_G_ = 0.31, and π_T_ = 0.18), and substitution rates (*r*_AC_ = 1.7, *r*_AG_ = 2.6, *r*_AT_ = 2.8, *r*_CG_ = 2.3, *r*_CT_ = 3.6, and *r*_GT_ = 1). Within the Bayesian phylogenetic inference, two chains converged at similar topologies. The standard deviation of split frequencies reached values lower than 0.01 during analysis, and the stationarity was reached after 2.27 million generations (Figure S1). The ML and the BI analyses indicated an identical phylogenetic pattern for *Sorghum* plus *Cleistachne sorghoides*.

**Figure 1:**
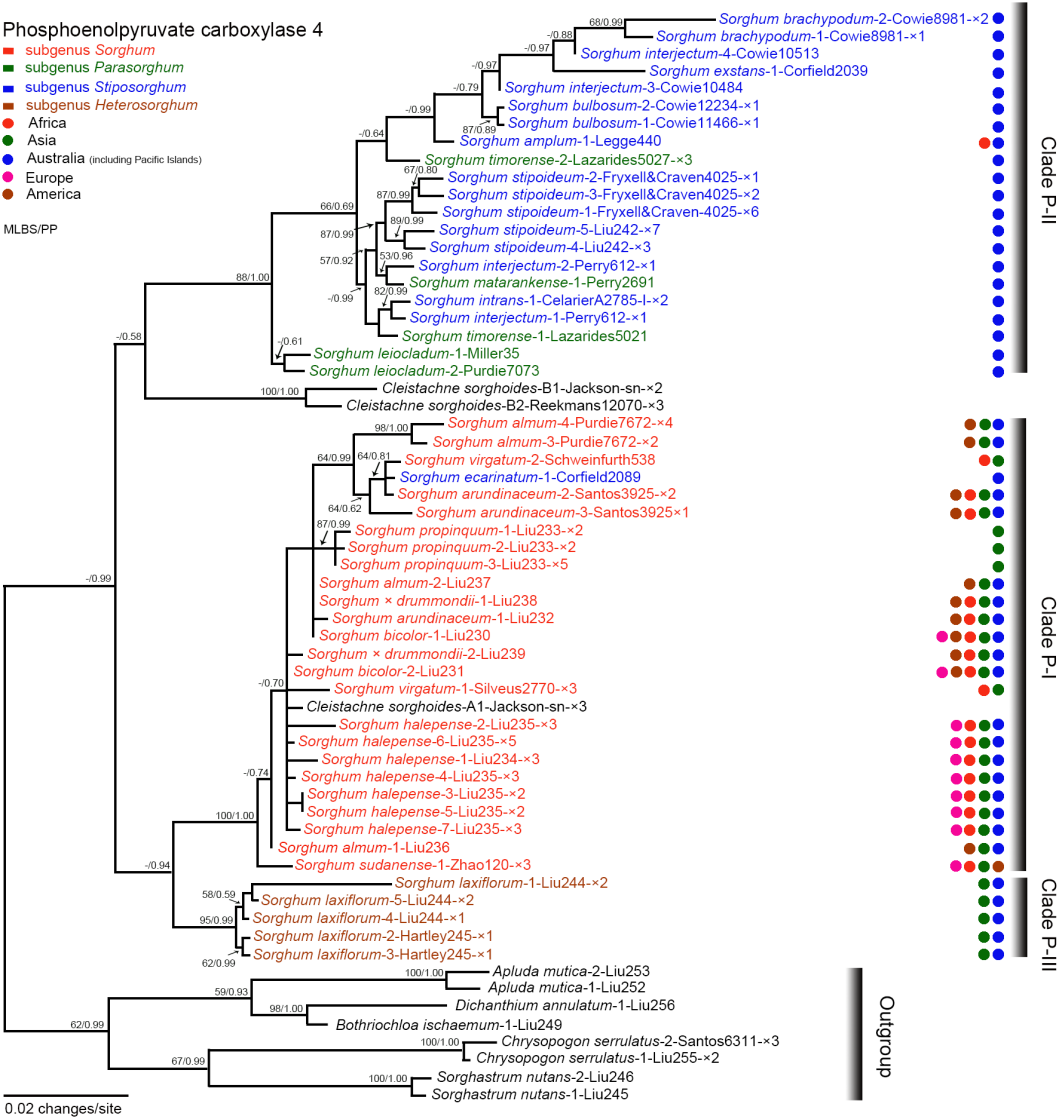
Maximum likelihood phylogeny of *Sorghum* inferred from nuclear *Pepc4* data. Numbers above branches are maximum likelihood bootstrap/Bayesian posterior probability (MLBS/PP). Taxon labels are in the format: *Sorghum brachypodum*-2-Cowie8981-×2 where *Sorghum brachypodum* indicates that the sequence belongs to the species *Sorghum brachypodum*; -2- = the second sequence listed in Table S1 for the species; Cowie8981 = specimen voucher information; -×2 indicates we recovered 2 clones for the sequence; and without any mark after specimen voucher information indicates the sequence is derived from PCR-direct sequencing. Coloured taxon labels and circles correspond to the listed subgenera and geographic ranges at the top left corner of the figure, respectively.

The monophyly of *Sorghum* plus *Cleistachne sorghoides* (with the latter nested within *Sorghum*) received strong support from the BI analysis (PP = 0.99). Three clades (designated as clades P-I, P-II, and P-III) were observed in the *Pepc4* phylogram with strong support (Figure 1). The *Pepc4* sequences from one accession of *Cleistachne sorghoides* fell into two divergent lineages [clade P-I and an independent branch with strong support (MLBP = 100%, PP = 1.00)], with clade P-I having A type sequence and the independent branch having B type sequences (putative homoeologues, a potential result caused by allotetraploidy, where each sequence type represents a different parental lineage). Clade P-I contained species of subg. *Sorghum*, *S. ecarinatum* Lazarides, and A-type sequence of *Cleistachne sorghoides* with strong support (MLBP = 100%, PP = 1.00). Clade P-II comprised subg. *Parasorghum* and *Stiposorghum* with strong or moderate support (MLBP = 88%, PP = 1.00). Clade P-III contained *S. laxiflorum* with strong support (MLBP = 95%, PP = 0.99). Clade P-I was sister to clade P-III (PP = 0.94), while clade P-II was sister to B-type sequences of *C. sorghoides* (PP = 0.58), and finally, the clade P-I+clade P-III was sister to the clade P-II and B-type sequences of *C. sorghoides* in the *Pepc4* phylogram (PP = 0.99) (Figure 1).

### Phylogenetic analyses of *GBSSI* sequences

The aligned *GBSSI* matrix comprised 1501 characters, including partial exons 8 and 13, complete exons 9, 10, 11, and 12, introns 8, 9, 10, 11, and 12 at a length of 82 bp, 33 bp, 185 bp, 204 bp, 106 bp, 138 bp, 158 bp, 152 bp, 145 bp, 130 bp, and 168 bp, respectively (Table 3). The log likelihood scores of 56 substitution models ranged from 11947.3877 to 12361.0693, and Modeltest indicates that the best-fit model under AIC is TIM+G with base frequencies (π_A_ = 0.23, π_C_ = 0.26, π_G_ = 0.28, and π_T_ = 0.23) and substitution rates (*r*_AC_ = 1.0, *r*_AG_ = 1.5, *r*_AT_ = 1.1, *r*_CG_ = 1.1, *r*_CT_ = 1.9, and *r*_GT_ = 1). Within the Bayesian phylogenetic inference, two chains converged at similar topologies. The standard deviation of split frequencies reached values lower than 0.01 during analysis, and stationarity was reached after 1.09 million generations (Figure S2). The ML and the BI analyses generated an identical phylogenetic pattern for *Sorghum*.

**Figure 2:**
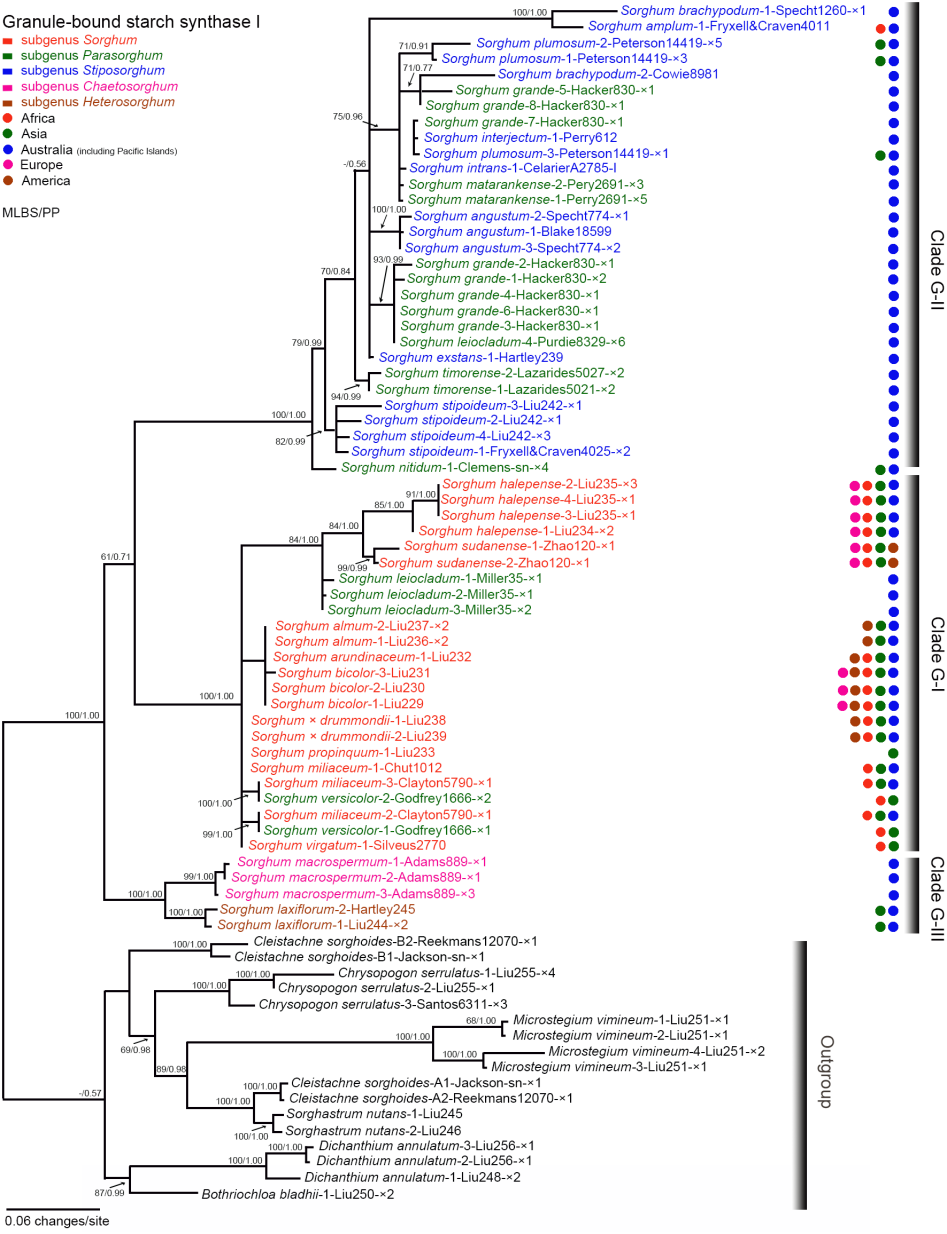
Maximum likelihood phylogeny of *Sorghum* inferred from nuclear *GBSSI* data. Numbers above branches are maximum likelihood bootstrap/Bayesian posterior probability (MLBS/PP). Taxon labels are in the format: *Sorghum matarankense*-2-Perry2691-×3 where *Sorghum matarankense* indicates that the sequence belongs to the species *Sorghum matarankense*; -2- = the second sequence listed in Table S1 for the species; Perry2691 = specimen voucher information; ×3 indicates we recovered 3 clones for the sequence; and without any mark after specimen voucher information indicates the sequence is derived from PCR-direct sequencing. Coloured taxon labels and circles correspond to the listed subgenera and geographic ranges at the top left corner of the figure, respectively.

The monophyly of *Sorghum* received strong support (MLBS = 100%, PP = 1.00) (Figure 2). Three clades (designated as clades G-I, G-II, and G-III) were recognized in the *GBSSI* phylogram with strong support. Clade G-I contained subg. *Sorghum* species, *S. leiocladum* (Hack.) C.E. Hubb., and *S. versicolor* Andersson with strong support (MLBP = 100%, PP = 1.00). Clade G-II comprised species of subg. *Parasorghum* and *Stiposorghum* with strong support (MLBP = 100%, PP = 1.00). Clade G-III consisted of *S. laxiflorum* and *S. macrospermum* with strong support (MLBP = 100%, PP = 1.00). Clade G-I was shown to be sister to clade G-II with weak support (MLBS = 0.61, PP = 0.71), and this group in turn, showed a strong association with clade G-III (MLBP = 100%, PP = 1.00) in the *GBSSI* phylogram (Figure 2).

Two (A- and B-type) homoeologous loci of *GBSSI* sequences were identified for two accessions of *Cleistachne sorghoides*, providing strong evidence for the presence of two divergent genomes. The A-type *GBSSI* sequences of *Cleistachne sorghoides* were characterized by three features: a large number of variations occurred in introns 8, 9, 11, and 12 (e.g., the strong support for A-type homoeologues of *C. sorghoides* and *Sorghastrum nutans* in Figure 1); the A-type homoeologues of *C. sorghoides* being distantly related to B-type homoeologues of *C. sorghoides* (Figure 2); and 13 insertions (3–17 bp in length) distributed in introns 8, 9, 11, and 12, implying the likelihood of sequence divergence after the speciation event of *C. sorghoides*.

### Divergence times

**Figure 3:**
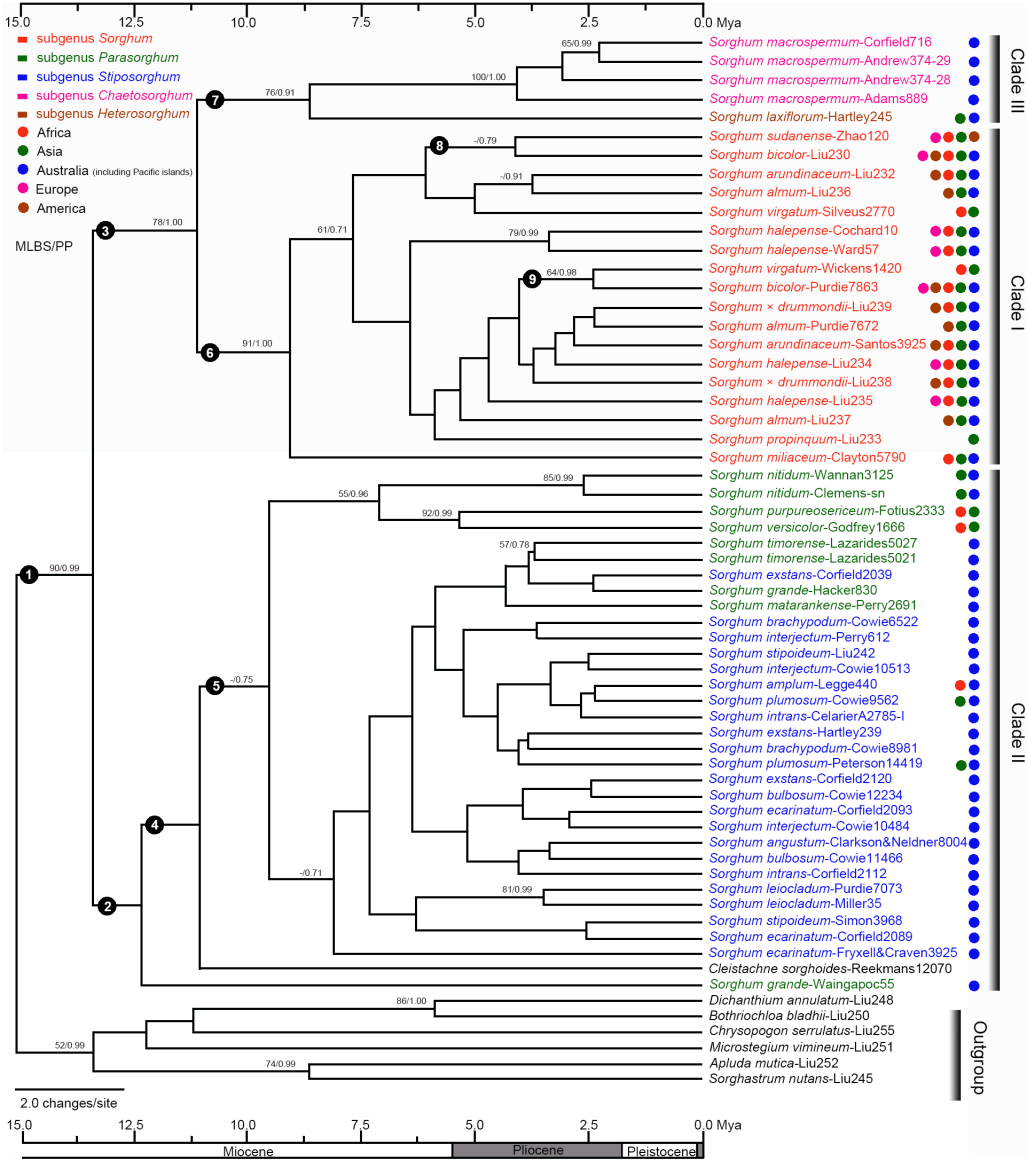
Chronogram of *Sorghum* and relatives based on three plastid sequences (*ndhA* intron, *rpl32-trnL*, and *rps16* intron) as inferred from BEAST. Numbers above the branches are maximum likelihood bootstrap/Bayesian posterior probability (MLBS/PP). Taxon labels are in the format: *Sorghum almum*-Liu236 where *Sorghum almum* indicates that the sequence belongs to the species *Sorghum almum*; -Liu236 = specimen voucher information. Coloured taxon labels and circles correspond to the listed subgenera and geographic ranges at the top left corner of the figure, respectively. Numbers 1–9 indicate the lineages of interest as shown in Table 4.

The combined plastid matrix of 62 accessions comprised 2858 characters, of which 113 were parsimony-informative (4.0%). The “cumulative” and “compare” results implemented in the AWTY showed that two runs had reached stationarity after 2.57 million generations (Figure S3). The BEAST analysis generated a well-supported tree (MLBP = 90%, PP = 0.99) for *Sorghum* plus *Cleistachne sorghoides* (Figure 3), which was identical to the topologies from ML and BI analyses. Three clades were recognized for *Sorghum* plus *Cleistachne sorghoides*. Clade II included *Cleistachne sorghoides* and subg. *Parasorghum* and *Stiposorghum* (lineage number 2), and clade I (i.e., subg. *Sorghum*) (lineage number 3) was sister to clade III (i.e., subg. *Chaetosorghum* and *Heterosorghum* ). Here we discuss divergence times for the lineages of interest as shown in Table 4.

**Table 4:** Posterior age distributions of lineages of interest in *Sorghum* plus *Cleistachne sorghoides*.

Lineage	N	Stem age (Mya)	Crown age (Mya)
*Sorghum* plus *Cleistachne sorghoides*	1	14.3 (5.6–18.0)	12.7 (5.5–16.7)
Clade II	2	12.7 (5.5–16.7)	11.7 (5.0–14.2)
Clades I+III	3	12.7 (5.5–16.7)	10.5 (4.1–14.1)
Clade II excluding *S. grande*	4	11.7 (5.0–14.2)	10.5 (4.1–13.8)
Clade II excluding *S. grande* and *Cleistachne sorghoides*	5	10.5 (4.1–13.8)	9.0 (3.3–11.5)
Clade I	6	10.5 (4.1–14.1)	8.6 (3.0–11.1)
Clade III	7	10.5 (4.1–14.1)	8.2 (2.3–11.1)
The *S. bicolor*-*S. sudanense* lineage (Africa, America, Asia, Europe)	8	5.8 (1.5–6.6)	3.9 (0.3–4.3)
The *S. bicolor*-*S. virgatum* lineage (Africa, Asia)	9	3.9 (0.1–4.0)	2.4 (0.0–3.4)

Lineage number (N) correspond to Figure 3; Lineage age is given by the mean age and the 95% highest posterior density (HPD) intervals in brackets; The age of each lineage is composed of the stem and the crown ages.

The uncorrelated-rates relaxed molecular clock suggests that the diversification of *Sorghum* plus *Cleistachne sorghoides* lineage occurred in the middle Miocene (12.7 Mya with 95% HPD of 5.5–16.7 Mya; lineage number 1 in Figure 3), which is the stem age for clade II (lineage number 2) and for clades I and III (lineage number 3). The crown age of clade II excluding *S. grande* was determined to be 10.5 (4.1–13.8) Mya in the late Miocene (lineage number 4), which is also the divergence time of clade II excluding *S. grande* and *Cleistachne sorghoides* (lineage number 5). The crown age of clade I was 10.5 (4.1–14.1) Mya in the late Miocene (lineage number 6), which is also the stem divergence time of clade III (lineage number 7) in Figure 3. Two lineages containing *S. bicolor* were estimated at 3.9 (0.3–4.3) Mya in the early Pliocene (the Africa-America-Asia-Europe lineage; lineage number 8) and 2.4 (0.0–3.4) Mya in the early Pliocene (the Africa-Asia lineage; lineage number 9), respectively (Table 4).

## Discussion

### Origin of *Cleistachne sorghoides*

Plastid, *Pepc4* and *GBSSI* data support the hypothesis for the allotetraploid origin of *Cleistachne sorghoides.* Based on the plastid data, *Cleistachne sorghoides* shared a common ancestor with clade II excluding *S. grande* (lineage number 4 in Figure 3), which may represent a source of the maternal parent for *C. sorghoides*. The plastid sequence similarity between *C. sorghoides* and clade II excluding *S. grande* also indicated that *C. sorghoides* became separated from the common ancestor in a relatively ancient time [10]. The *Pepc4* data provide evidence for this ancient allopolyploid origin because the conservative *Pepc4* gene evolved more slowly than non-housekeeping genes [75]. Two *Pepc4* homoeologous loci of *C. sorghoides* were isolated from the same accession, and this indicates the presence of two divergent genomes in *C. sorghoides*. The maternal lineage identified by the plastid tree was confirmed by the weak relationship between clade P-II and B-type homoeologues of *C. sorghoides* in the *Pepc4* phylogeny (Figure 1). The *GBSSI* tree was found to be complementary to the nrDNA ITS tree, in which *C. sorghoides* was deeply nested within the subg. *Parasorghum* and *Stiposorghum* lineage [8]. The authors inferred that the ITS sequences of *C. sorghoides* might have undergone complete homogenization towards the maternal parent, i.e. the subg. *Parasorghum* and *Stiposorghum* lineage. The B-type homoeologues of *Cleistachne sorghoides* showed no close relationship with any sampled species in the *GBSSI* tree (Figure 2), providing indirect evidence for the full divergence of B-type *GBSSI* homoeologues of *C. sorghoides* away from the maternal parent in *Sorghum* (clade II) in the *GBSSI* tree.

The paternal parent of *Cleistachne sorghoides* remains unresolved due to the incongruence between the two LCN trees. In the *Pepc4* tree, A-type homoeologue of *C. sorghoides* shared a common ancestor with clade P-I native to the Old World, while A-type *GBSSI* homoeologues of *C. sorghoides* showed a strong relationship with *Sorghastrum nutans* in the *GBSSI* tree. Considering its geographic range in North America, *Sorghastrum nutans* seems a much less likely candidate as the paternal parent for *C. sorghoides* because geographically there is no opportunity for sexual contact with its potential maternal lineage.

To explain the paternal genome of *Cleistachne sorghoides*, it seems likely that *C. sorghoides* acquired the A-type *Pepc4* sequences via hybridization with the ancestor of subg. *Sorghum*, and subsequently the A-type *GBSSI* sequences of *C. sorghoides* experienced recombination (gene exchange) with species of the of African-American disjunct *Sorghastrum* [11]. A pre-requisite of this hypothesis is that East Africa and India would have been the geographic location of the recombination episode, perhaps in the fallow lands of Sudan, Uganda, Kenya, Congo, and India, where the native distribution of *C. sorghoides* is found [11]. Therefore, the recombination event of *C. sorghoides* placed its *GBSSI* homoeologues near the outgroup location in the *GBSSI* phylogram. The LCN data indicate that *C. sorghoides* may have experienced a complex speciation process [2]. Based on support from *Pepc4*, combined plastid, and previous restriction site data [76], we chose to transfer *Cleistachne sorghoides* into *Sorghum* (Table 5).

**Table 5:** A proposed new subgeneric classification of *Sorghum* Moench (subtribe Sorghinae Clayton & Renvoize, tribe Andropogoneae Dumort.) based on plastid and nuclear DNA data (*not examined in this study).

subg. ***Chaetosorghum*** E.D. Garber
sect. ***Chaetosorghum*** (E.D. Garber) Ivanjuk. & Doronina
*S. macrospermum* E.D. Garber
sect. ***Heterosorghum*** (E.D. Garber) Ivanjuk. & Doronina
*S. laxiflorum* F.M. Bailey
subg. ***Parasorghum*** (Snowden) E.D. Garber [syn: subg. *Stiposorghum* E.D. Garber]
*S. amplum* Lazarides, *S. angustum* S.T. Blake, *S. bulbosum* Lazarides, *S. brachypodum* Lazarides, *S. ecarinatum* Lazarides, *S. exstans* Lazarides, *S. grande* Lazarides, *S. interjectum* Lazarides, *S. intrans* F. Muell. ex Benth., *S. leiocladum* (Hack.) C.E. Hubb., *S. matarankense* E.D. Garber & L.A. Snyder, *S. nitidum* (Vahl) Pers., *S. plumosum* (R.Br.) P. Beauv., *S. purpureosericeum* (Hochst. ex A. Rich.) Asch. & Schweinf., *S. stipoideum* (Ewart & Jean White) C.A. Gardner & C.E. Hubb., *S. timorense* (Kunth) Büse, *S. versicolor* Andersson
subg. ***Sorghum***
* S. almum* Parodi, *S. arundinaceum* (Desv.) Stapf, *S. bicolor* (L.) Moench, *S.* x *drummondii* (Nees ex Steud. ) Millsp. & Chase, *S. halepense* (L.) Pers., *S. miliaceum* (Roxb.) Snowden, *S. propinquum* (Kunth) Hitchc., *S. sudanense* (Piper) Stapf, *S. virgatum* (Hack.) Stapf
Incertae sedis
*S. burmahicum Raizada, *S. controversum (Steud.) Snowden, *S. x *derzhavinii* Tzvelev, *S. sorghoides* (Benth.) Q. Liu & P.M. Peterson,*S. trichocladum (Rupr. ex Hack.) Kuntze

### Infrageneric phylogenetic relationships in *Sorghum*

The monophyly of *Sorghum* plus *Cleistachne sorghoides* is supported by *Pepc4* and plastid data, as well as the combined ITS1/*ndhF*/*Adh1* data [14], where *Sorghum* plus *Cleistachne sorghoides* are resolved into a distinct clade with 100% support. Nevertheless, the result contradicts the monophyly of *Sorghum* supported by *GBSSI* data. The absence of a definitive boundary for members of the subtribe Sorghinae has led others to suggest that the subtribe might have experienced rapid radiation [41]. The gene recombination event was inferred to explain the *GBSSI* sequence divergence of *C. sorghoides* from *Sorghum*, thus the unresolved phylogenetic position of the B-type *GBSSI* homoeologues of *C. sorghoides* in the *GBSSI* tree may indicate a complex phylogenetic history of the Sorghinae.

Three infrageneric lineages were supported by the LCN and the plastid data: the subg. *Sorghum* lineage; the subg. *Parasorghum* and *Stiposorghum* lineage; and the subg. *Chaetosorghum* and *Heterosorghum* lineage. The subg. *Chaetosorghum* and *Heterosorghum* lineage contained *S. macrospermum* and *S. laxiflorum*, respectively (Figures 2 and 3). These two species were easily distinguished from the remaining Australian native species of *Sorghum* in having glabrous culm nodes, reduced pedicelled spikelets, and a minute obtuse callus [2], [3]. The two species possessed relatively smaller 2C DNA content (2.07 pg to 2.49 pg) than the remaining congeneric Australian species [3], [36], [77], [78]. The close relationship between *S. macrospermum* and *S. laxiflorum* was also supported by nrDNA ITS [8], [10] and the combined ITS1/*ndhF*/*Adh1* [9], [14], On the basis of morphological, cytogenetic, and molecular sequence evidence, it is appropriate to recognize a distinct subg. *Chaetosorghum* comprising two sections: sect. *Chaetosorghum* (E.D. Garber) Ivanjuk. & Doronina (*S. macrospermum*) and sect. *Heterosorghum* (E.D. Garber) Ivanjuk. & Doronina (*S. laxiflorum*) (Table 5), although we could not get clean *Pepc4* sequences of *S. macrospermum* in the laboratory.

Most species of subg. *Parasorghum* and *Stiposorghum* were resolved into one well-supported lineage in the two LCN phylograms. The two subgenera were traditionally distinguished by length and shape of the callus on the sessile spikelet: *Parasorghum* was characterized by a short and blunt callus with an articulation joint, whereas *Stiposorghum* was characterized by a long and pointed callus with a linear joint [2], [3]. However, doubts have recently been cast on the systematic value of the callus owing to the continuity of character states across the subgeneric boundary [14]. The subjective nature of determining callus morphology was also reflected by the molecular results because members of *Parasorghum* and *Stiposorghum* were aligned into a single lineage [7], [8], [40]. Since there were no well-defined taxonomic and genetic boundaries between these two subgenera, the most practical solution is to combine them into a single subg. *Parasorghum* (Table 5).

Subgenus *Chaetosorghum* (including *S. macrospermum* and *S. laxiflorum*) appears closely related to subg. *Sorghum* with strong support (PP = 1.00) in the plastid tree (Figure 3); and such a relationship is consistent with nrDNA ITS [8], the combined ITS1/*ndhF*/*Adh1* [14], and *Pepc4* sequence data (Figure 1). Although the relationship between subg. *Chaetosorghum* and the clade G-I+clade G-II lineage received weak support (MLBS = 0.61, PP = 0.71) in the *GBSSI* tree, the placement of subg. *Chaetosorghum* in *Sorghum* is unequivocally supported by the sequence data [79].

### Interspecific relationships within subg. *Sorghum* and GP-3 species

In the *Pepc4* phylogram, weak support (MPBS<50%, PP<0.5) was found for *S. bicolor* (Australian and Mexican accessions) and its immediate wild relatives, i.e., *S. almum*, *S. arundinaceum*, *S.* x *drummondii*, *S. propinquum*, and *S. virgatum* (Figure 1). The five species formed a strongly supported clade G-I (Figure 2). Based on the short branch lengths within clade P-I and clade G-I, the ease to hybrid formation between *S. bicolor* and certain members of subg. *Sorghum* [80], and their similar karyotypes [81], it is reasonable to infer that the ancestors of *S. bicolor* may be members of subg. *Sorghum* [82]. It was suggested that *S. almum* was a recent fertile hybrid between *S. bicolor* and *S. halepense* [80], but *S. arundinaceum*, *S. bicolor*, *S.* x *drummondii*, *S. propinquum*, and *S. virgatum* appear closely related to *S. almum* in *Pepc4*, *GBSSI*, and plastid phylograms, suggesting that they may be potential genome donors to *S. almum* [16].

*Sorghum bicolor* is an annual diploid species native to Africa [13]. Four main hypotheses have been proposed to explain its early evolutionary history: (1) annual *S. arundinaceum* was assumed to be the wild progenitor of *S. bicolor* based on a cytological study [11]; (2) *S. bicolor* was thought to be an interspecific hybrid and a descendant of two diploid species (2*n* = 10) [83]; (3) *S. bicolor* may have arisen by chromosome doubling from one diploid ancestor (2*n* = 10) [84]; or (4) *S. bicolor* may share a common ancestor with sugarcane and maize through an ancient polyploidization event [85]. The first hypothesis is supported by our study, where *S. arundinaceum* is confirmed to have a close relationship with *S. bicolor*, and this is seen in our LCN trees. Being an ancient forest-savanna species native to tropical Africa [86], *Sorghum arundinaceum* extends eastwards to India, Australia, and is introduced to tropical America [5], [11]. It is possible that the cultivated sorghum originated from *S. arundinaceum* native to forest-savanna in the sub-Saharan belt at the north of the equator before it colonized regions from the Atlantic to the Indian Oceans.

The separation of *S. sudanense* (Sudan grass) from *S.* x *drummondii* is supported by our study. The two species are distributed from Sudan to Egypt in East Africa [13] and naturalized in China and the Americas [39]. The relationship between these two species was incongruent based on the two LCN gene phylograms. The *Pepc4* sequences suggest that *S. sudanense* is sister to the lineage containing *S.* x *drummondii* and the remainder of subg. *Sorghum* with strong support (MLBS = 100%, PP = 1.00, Figure 1), it appears that *S. sudanense* is genetically distant from *S.* x *drummondii*. While in the *GBSSI* phylogram, the two species are nested within a strongly supported clade G-I (MLBS = 100%, PP = 1.00, Figure 2). An interpretation of the incongruent pattern might be that *S. sudanense* was a consequence of sympatric speciation among different East African populations of *S.* x *drummondii* occurring abundant genetic variation [87]. *Sorghum sudanense* has obovate caryopses with smooth surfaces whereas *S.* x *drummondii* has obovate or elliptic caryopses with striate surfaces (H. Liu et al., unpublished data). Perhaps caryopses with different surface sculptures are the phenotypic consequence of adaptation to different microhabitats [88], [89]. Recognition of the two taxa at the specific level, as opposed to merging them as varieties [13] is compatible with our results.

The genome origin of *S. halepense* has been debated for years. It was believed that *S. halepense* experienced homoeologous chromosome transpositions [90] from potential progenitors *S. bicolor* and *S. propinquum* [91], [92]. Some workers proposed that *S. halepense* was a segmental allotetraploid hybrid between *S. arundinaceum* and *S. propinquum* [12], [80]. If so, the maternal parents of *S. halepense* may have come from members of subg. *Sorghum*, since *S. halepense* is deeply nested within lineage number 6 (Figure 3). Furthermore, the plastid data supports *S. arundinaceum* and *S.* x *drummondii* as potential progenitors of *S. halepense*. An alternative hypothesis is that *S. halepense* is an interspecific hybrid and a descendant of *S. bicolor* and *S. virgatum* [93]. However, the *Pepc4* and *GBSSI* data contradict this hypothesis since no corresponding loci were isolated from *S. halepense*. In *GBSSI* tree, four sequences of *S. halepense* formed a lineage (MLBS = 85%, PP = 1.00), which was sister to the *S. sudanense* lineage. These results are consistent with the hypothesis that *S. halepense* arose via homoeologous chromosome transpositions from members of subg. *Sorghum*. *Sorghum halepense* exhibits disomic inheritance [38], [83], allowing the independent assortment of DNA segments between progenitors resulting in a complex evolutionary pattern [94]. This assumption is substantiated in allozyme studies, where high-frequency alleles found in *S. halepense* were not detected in *S. bicolor* or *S. propinquum*, providing further evidence for the absence of alleles from progenitors of *S. halepense* [95].

Based on *GBSSI* and plastid data, *Sorghum nitidum* is nested within the subg. *Parasorghum* and *Stiposorghum* lineage. *Sorghum nitidum* is distributed in southeast Asia, the Pacific Islands, and northern Australia [2], and exhibits significant morphological variation. The species is characterized by a hairy ring around the nodes, awnless or awned lemmas in sessile spikelets, and relatively small chromosomes [81]. Based on ITS and *ndhF* analyses, *S. nitidum* is embedded in subg. *Sorghum* [16]. However, the genome size of *S. nitidum* (2.20 pg) resembles that of members of subg. *Parasorghum* and *Stiposorghum* (0.64 pg–2.30 pg) rather than that of subg. *Sorghum* (0.26 pg–0.42 pg) [36]. Our study supports a close relationship between *S. nitidum* and the subg. *Parasorghum* and *Stiposorghum* lineage [2], [9].

### Palaeoclimatic hypothesis for lineage divergence in *Sorghum*

It is recognized that the evolution of organisms is profoundly influenced by past tectonic activities and climate changes [30], [96]. Two *Sorghum* major lineages (lineage numbers 2 and 3) diverged from a common ancestor at 12.7 (95% HPD: 5.5–16.7) Mya (Figure 3) in the middle Miocene-Pliocene interval marked by aridification, which induced C_4_ grassland emergences in Africa [28], [97]. The Eastern branch of East Africa Rift has continuously uplifted since the early Miocene [98], [99], and the increasingly arid climate of tropical and subtropical Africa was caused by the topographic barrier of the eastern branch Rift to moist maritime air from the Indian Ocean [100], [101]. The resultant formation of new ecological niches [28] presumably catalyzed the diversification of *Sorghum* (e.g., lineage numbers 8 and 9 in Figure 3) in Africa at a time when significant faunal turnover was observed, e.g., leaf-mining flies [102], savanna-inhabiting crickets [103], prairie-adapted rodents [104], and grass-feeding mammals [105].

The northern Australian endemic species of *Sorghum* (mostly in lineage number 5, Figure 3) diverged by 9.0 (HPD: 3.3–11.5) Mya around the late Miocene/Pliocene boundary, when the monsoonal palaeoclimate was characterized by south-eastward dry trade winds in winter and north-westward moist flow in summer [106]–[108]. The Australian endemic species [e.g., *S. intrans*, *S. leiocladum*, *S. matarankense* E.D. Garber & L.A. Snyder, and *S. timorense* (Kunth) Büse] are geographically restricted to rocky hills, coastal dunes, and seasonally flooded swamps in northern Australia [3], [5] where the local vegetation was affected by the lowering seas, leading to the dominance of monsoonal savannas [109]. Meanwhile, the highly dissected tropical areas became even more scattered in northern Australia causing complex topography in the monsoonal savannas. Therefore, it is reasonable to hypothesize that the dominance of monsoonal savanna in the late Miocene contributed to the high level of endemism of *Sorghum* in Australia.

### Taxonomy

Traditionally, *Cleistachne* has been separated from *Sorghum* because it has only single spikelets whose pedicels are thought to represent raceme peduncles, whereas *Sorghum* has sessile and pedicelled spikelets, although the sessile spikelets can be much reduced [6], [11]. Our study and that of early workers agree that *Cleistachne* is allied with *Sorghum* [6], [11], [110]; we thus propose the new combination as below.

***Sorghum sorghoides*** (Benth.) Q. Liu & P.M. Peterson, ***comb. nov*** *.* Basionym: *Cleistachne sorghoides* Benth., Hooker’s Icon. Pl. 14: t. 1379. 1882.

We also propose a new subgeneric classification of *Sorghum* (Table 5). Within *Sorghum* we recognized three subgenera: *Chaetosorghum*, *Parasorghum*, and *Sorghum*; and chose to retain two sections within *Chaetosorghum*: *Chaetosorghum* and *Heterosorghum*. Alternatively, based on our molecular results, one could use the new generic name *Sarga* to represent species in subg. *Parasorghum, Sorghum* for species in subg. *Sorghum, Vacoparis* for species in *Chaetosorghum* and retain *Cleistachne*. Perhaps with a greater number of molecular markers, the apparent hybrid origin of *S. sorghoides* and phylogenetic position of *S. burmahicum* Raizada, *S. controversum* (Steud.) Snowden, *S. derzhavinii* Tzvelev, and *S. trichocladum* (Rupr. ex Hack.) Kuntze (all incertae sedis in our classification) will be elucidated.

### Conclusions

The monophyly of *Sorghum* plus *Cleistachne sorghoides* is supported by the *Pepc4* and the plastid data, and we provide a new combination, *Sorghum sorghoides*. Molecular results support the allotetraploid origin of *S*. *sorghoides*. Based on combined plastid data, members of subg. *Parasorghum* may represent the maternal parents, while the paternal parents of *S*. *sorghoides* remained unresolved because of incongruence between the *Pepc4* and the *GBSSI* phylograms. *Sorghum macrospermum* is sister to *S. laxiflorum*, forming a distinct clade, which we refer to as subg. *Chaetosorghum* with two sections *Chaetosorghum* (*S. macrospermum*) and *Heterosorghum* (*S. laxiflorum*). Most of members of the two subgenera *Parasorghum* and *Stiposorghum* are resolved into one well-supported lineage by the two LCN phylograms. Therefore, we choose to recognize a single subg. *Parasorghum*, and place *Stiposorghum* in synonymy. The two LCN gene trees and the combined plastid tree are consistent with the hypothesis that *S. halepense* originated via homoeologous chromosome transpositions. During the middle Miocene-Pliocene interval, the formation of new ecological niches in tropical and subtropical Africa presumably catalysed the diversification of *Sorghum* in Africa. Furthermore, it seems reasonable to infer that the dominance of monsoonal savanna in the late Miocene contributed to the high level of endemism of *Sorghum* in Australia. Molecular results support the recognition of three distinct subgenera in *Sorghum*: subg. *Chaetosorghum* with two sections each containing a single species, subg. *Parasorghum* with 17 species, and subg. *Sorghum* with nine species.

## Supporting Information

Figure S1 **Results of the exploration of** ***Pepc4*** **MCMC convergence using the AWTY (Are We There Yet?) approach.** (a) Cumulative plot of the posterior probabilities of 20 splits at selected increments over one of two MCMC runs. (b) Comparative plot of posterior probabilities of all splits for paired MCMC runs.
(TIF)

Figure S2 **Results of the exploration of** ***GBSSI*** **MCMC convergence using the AWTY (Are We There Yet?) approach.** (a) Cumulative plot of the posterior probabilities of 20 splits at selected increments over one of two MCMC runs. (b) Comparative plot of posterior probabilities of all splits for paired MCMC runs.
(TIF)

Figure S3 **Results of the exploration of three plastid sequences** ***(ndhA*** **intron,** ***rpl32-trnL*** **and** ***rps16*** **intron** ***)*** **MCMC convergence using the AWTY (Are We There Yet?) approach.** (a) Cumulative plot of the posterior probabilities of 20 splits at selected increments over one of two MCMC runs. (b) Comparative plot of posterior probabilities of all splits for paired MCMC runs.
(TIF)

Table S1 **Taxon name, chromosome number, source, and GenBank accession numbers of** ***Pepc4*** **,** ***GBSSI*** **, and three plastid (** ***ndhA*** **intron,** ***rpl32-trnL*** **, and** ***rps16*** **intron) sequences used in the study.**
(DOCX)
