# Supplementary figures and images for: Infrageneric Phylogeny and Temporal Divergence of Sorghum (Andropogoneae, Poaceae) Based on Low-Copy Nuclear and Plastid Sequences
Source: PLoS One. 2014 Aug 14;9(8):e104933. doi: 10.1371/journal.pone.0104933 (PMC4133246; doi:10.1371/journal.pone.0104933)

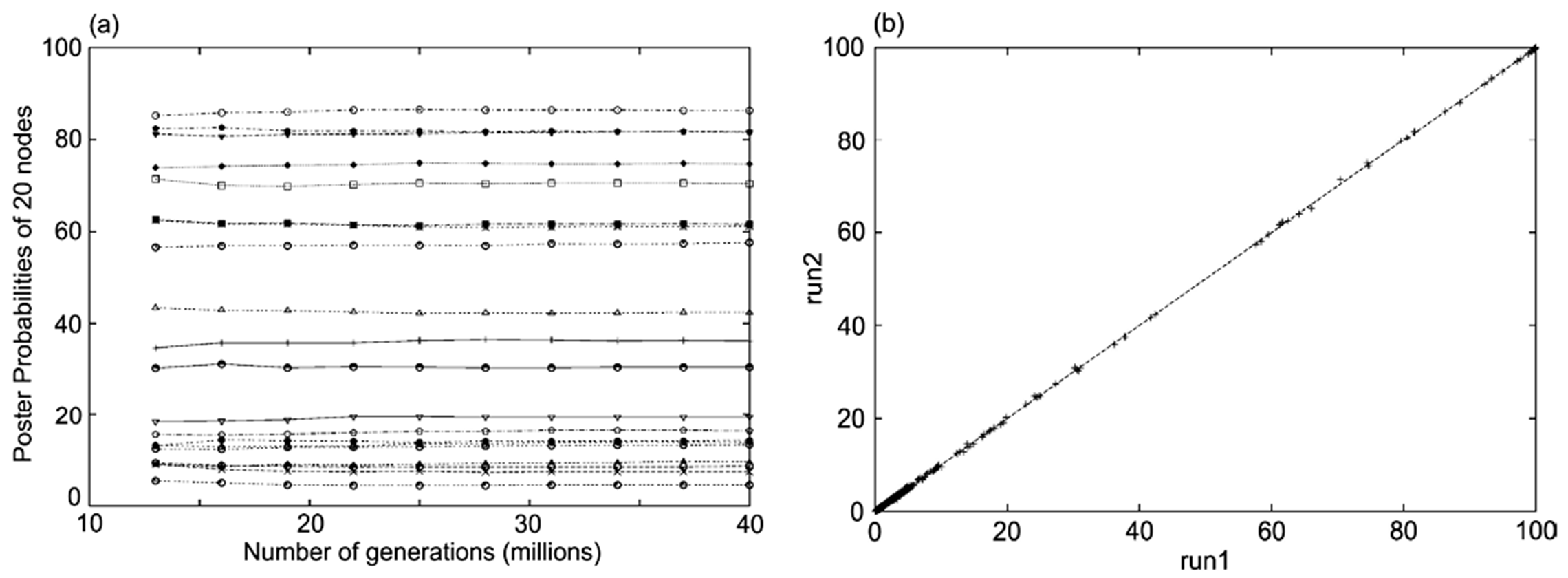

Supplement: Figure S1 — Results of the exploration of Pepc4 MCMC convergence using the AWTY (Are We There Yet?) approach. (a) Cumulative plot of the posterior probabilities of 20 splits at selected increments over one of two MCMC runs. (b) Comparative plot of posterior probabilities of all splits for paired MCMC runs. (TIF) [file pone.0104933.s001.tif]

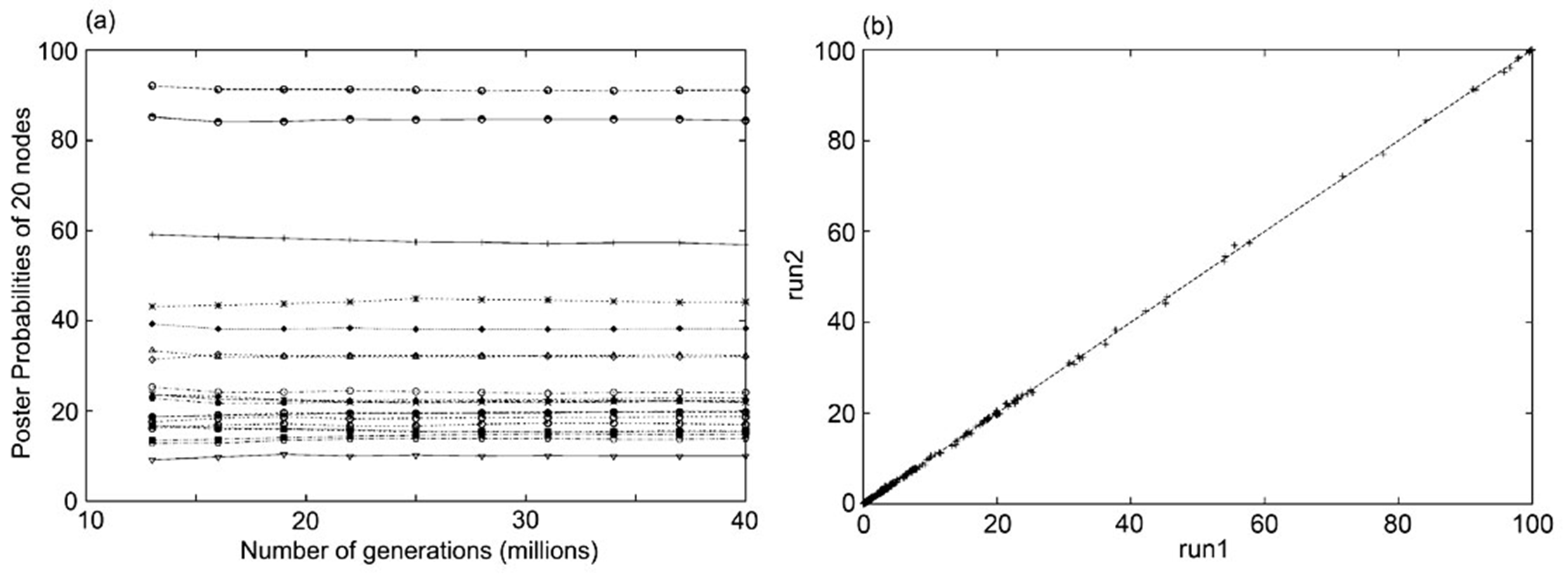

Supplement: Figure S2 — Results of the exploration of GBSSI MCMC convergence using the AWTY (Are We There Yet?) approach. (a) Cumulative plot of the posterior probabilities of 20 splits at selected increments over one of two MCMC runs. (b) Comparative plot of posterior probabilities of all splits for paired MCMC runs. (TIF) [file pone.0104933.s002.tif]

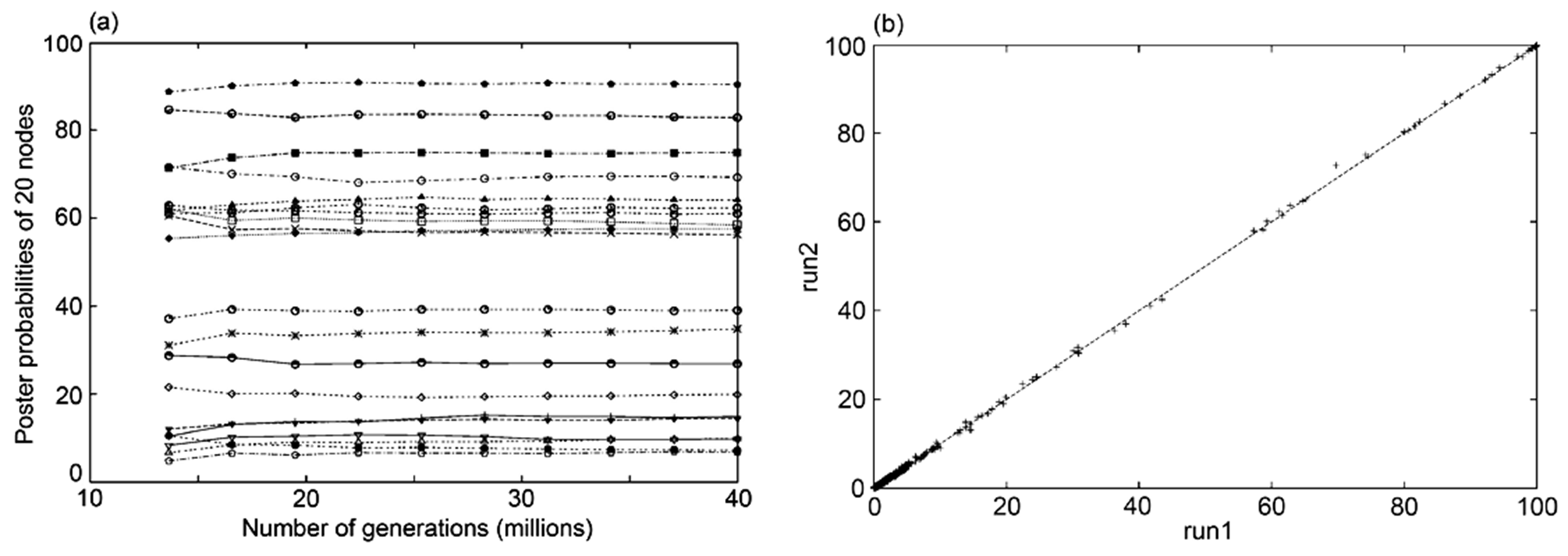

Supplement: Figure S3 — Results of the exploration of three plastid sequences (ndhA intron, rpl32-trnL and rps16 intron ) MCMC convergence using the AWTY (Are We There Yet?) approach. (a) Cumulative plot of the posterior probabilities of 20 splits at selected increments over one of two MCMC runs. (b) Comparative plot of posterior probabilities of all splits for paired MCMC runs. (TIF) [file pone.0104933.s003.tif]
