# Supplementary material for: Infrageneric Phylogeny and Temporal Divergence of Sorghum (Andropogoneae, Poaceae) Based on Low-Copy Nuclear and Plastid Sequences
Source: PLoS One. 2014 Aug 14;9(8):e104933. doi: 10.1371/journal.pone.0104933 (PMC4133246; doi:10.1371/journal.pone.0104933)
Supplement: Table S1 — Taxon name, chromosome number, source, and GenBank accession numbers of Pepc4 , GBSSI , and three plastid ( ndhA intron, rpl32-trnL , and rps16 intron) sequences used in the study. (DOCX) [file pone.0104933.s004.docx]

**Table S1** Taxon name, chromosome number, source, and GenBank accession numbers of *Pepc4*, *GBSSI*, and three plastid gene (*ndhA* intron, *rpl32-trnL*, and *rps16* intron) sequences used in the study.

| Subgenus *Sorghum* |
| --- |
| *Sorghum almum* Parodi (2*n* = 40) |
| Liu 236 (ILRI 16624); IBSC and US; Namibia; (1 KC428171; **1 KC469392** ×2); (KC428215; KC428278; KC428330) |
| Liu 237 (ILRI 13515); IBSC and US; Ethiopia; (2 KC428172; **2 KC469393** ×2); (KC428216; KC428279; KC428358) |
| Purdie 7672; CANB; Australia; (**3 KJ661126** ×2, **4 KJ661127** ×4; -); (-; KJ661164; KJ661191) |
| *Sorghum arundinaceum* (Desv.) Stapf (2*n* = 20) |
| Liu 232 (ILRI 13283); IBSC and US; Kenya; (1 KC428179 ; 1 KC469388); (KC428209; KC428291; KC428352) |
| Santos 3925; K; Brazil; (**2 KJ661129** ×2, **3 KJ661130** ×1; -); (-; KJ661165; KJ661192) |
| *Sorghum bicolor* (L.) Moench (2*n* = 20) |
| Liu 229 (IS 1); IBSC; Mexico; (-; 1 KC469382); (-; -; -)  Liu 230 (PI 17548); IBSC and US; Australia; (1 KC428178; 2 KC469383); (KC428214; KC428289; KC428335) |
| Liu 231 (IS 11); IBSC and US; Mexico; (2 KC428174; 3 KC469384); (-; -; -) |
| Purdie 7863; CANB; Australia; (-; -); (-; KJ661166; KJ661193) |
| *Sorghum × drummondii* (Nees ex Steud.) Millsp. & Chase (2*n* = 20) |
| Liu 238 (ILRI 15974); IBSC and US; China; (1 KC428176; 1 KC469395); (KC428205; KC428293; KC428344) |
| Liu 239 (ILRI 13333); IBSC and US; Kenya; (2 KC428173; 2 KC469396); (KC428206; KC428294; KC428345) |
| *Sorghum halepense* (L.) Pers. (2*n* = 40) |
| Liu 234 (PI 302268); IBSC and US; Tanzania; (**1 KC428147** ×3; **1 KC469360** ×2); (KC428207; KC428295; KC428337)  Liu 235 (PI 302281); IBSC and US; Australia; (**2 KC428149** ×3, **3 KC428161** ×2, **4 KC428164** ×3, **5 KC428165** ×2, **6 KC428166** ×5, **7 KC428170** ×3; **2 KC469362** ×3, **3 KC469363** ×1, **4 KC469364** ×1); (KC428208; KC428296; KC428338) |
| Cochard 10; CANB; Australia; (-; -); (-; KJ661167; KJ661194) |
| Ward 57; CANB; Australia; (-; -); (-; KJ661168; KJ661195) |
| *Sorghum miliaceum* (Roxb.) Snowden (2*n* =20) |
| Chut 1012; US; Australia; (-; 1 KC469385); (-; -; -) |
| Clayton 5790; US; Sri Lanka; (-; **2 KC469386** ×1, **3 KC469387** ×1); (-; KC428284; KC428351) |
| *Sorghum propinquum* (Kunth) Hitchc. (2*n* = 20) |
| Liu 233 (PI 653737); IBSC and US; USA; (**1 KC428152** ×2, **2 KC428153** ×2, **3 KC428155** ×5; 1 KC469394); (KC428217; KC428285; KC428346) |
| *Sorghum sudanense* (Piper) Stapf (2*n* = 20) |
| Zhao 120; IBSC; China; (**1 KC428146** ×3; **1 KC469358** ×1, **2 KC469359** ×1); (KC428219; KC428277; KC428340) |
| *Sorghum virgatum* (Hack.) Stapf (2*n* = 20) |
| Silveus 2770; US; USA; (**1 KC428150** ×3; 1 KC469381); (KC428212; KC428286; KC428332)  Schweinfurth 538; K; Sudan; (2 KJ661128; -); (-; -; -) |
| Wickens 1420; K; Sudan; (-; -); (-; KJ661169; KJ661196) |
| Subgenus *Parasorghum* |
| *Sorghum grande* Lazarides (2n = 30/40) |
| Hacker 830; CANB; Australia; (-; **1 KJ661146** ×2, **2 KJ661147** ×1, **3 KJ661148** ×1, **4 KJ661149** ×1, **5 KJ661151** ×1, **6 KJ661152** ×1, **7 KJ661153** ×1, **8 KJ661154** ×1); (-; -; KJ661201) |
| Waingapoc 55; US; Australia; (-; -); (KC428220; KC428263; KC428325) |
| *Sorghum leiocladum* (Hack.) C.E. Hubb. (2*n* = 20) |
| Miller 35; US; Australia; (1 KC428109; **1 KC469371** ×1, **2 KC469372** ×1, **3 KC469373** ×2); (KC428229; KC428270; KC428350) |
| Purdie 7073; CANB; Australia; (2 KJ661134; -); (-; KJ661174; KJ661202) |
| Purdie 8329; CANB; Australia; (-; **4 KJ661150** ×6); (-; -; -) |
| *Sorghum matarankense* E.D. Garber & L.A. Snyder (2n = 10) |
| Perry 2691; US; Australia; (1 KC428135; **1 KC469330** ×5, **2 KC469334** ×3); (KC428239; KC428274; KC428368) |
| *Sorghum nitidum* (Vahl) Pers. (2*n* = 20/rarely 10) |
| Clemens s. n.; US; Australia; (-; **1 KC469326** ×4); (KC428237; KC428269; KC428369) |
| Wannan 3152; CANB; Australia; (-; -); (-; KJ661175; KJ661203) |
| *Sorghum purpureosericeum* (Hochst. ex A. Rich.) Asch. & Schweinf. (2*n* = 10) |
| Fotius 2333; K; Cameroon; (-; -); (-; KJ661176; KJ661204) |
| *Sorghum timorense* (Kunth) Büse (2n = 10/rarely 20) |
| Lazarides 5021; US; Australia; (1 KC428110; **1 KC469348** ×2); (KC428235; KC428272; KC428355) |
| Lazarides 5027; US; Australia; (**2 KC428111** ×3; **2 KC469347** ×2); (KC428236; KC428273; KC428357) |
| *Sorghum versicolor* Andersson (2*n* = 10) |
| Godfrey 1666; US; South Africa; (-; **1 KC469367** ×1, **2 KC469368** ×2); (KC428203; KC428268; KC428327) |
| Subgenus *Stiposorghum* |
| *Sorghum amplum* Lazarides (2*n* = 10/30) |
| Fryxell & Craven 4011; US; Australia; (-; 1 KC469322); ( -; -; -) |
| Legge 440; CANB; Australia; (1 KJ661140; -); (KJ661157; KJ661177; KJ661205) |
| *Sorghum angustum* S.T. Blake (2n = 10) |
| Blake 18599; US; Australia; (-; 1 KC469344); (-; -; -)  Specht 774; US; Australia; (-; **2 KC469343** ×1, **3 KC469345** ×2); (-; -; -) |
| Clarkson & Neldner 8004; K; Australia (-; -); (-; -; KJ661206) |
| *Sorghum brachypodum* Lazarides (2*n* = 10) |
| Specht 1260; US; Australia; (-; **1 KJ661156** ×1); (-; -; -) |
| Cowie 6522; CANB; Australia; (-; -); (-; KJ661178; KJ661207) |
| Cowie 8981; CANB; Australia; (**1 KJ661132** ×1, **2 KJ661133** ×2; 2 KJ661155); (KJ661158; KJ661179; KJ661208) |
| *Sorghum bulbosum* Lazarides (2*n* = 10) |
| Cowie 11466; CANB ; Australia; (**1 KJ661135** ×1; -); (-; KJ661181; KJ661210) |
| Cowie 12234; CANB ; Australia; (**2 KJ661136** ×1; -); (KJ661159; KJ661180; KJ661209) |
| *Sorghum ecarinatum* Lazarides (2*n* = 10) |
| Fryxell & Craven 3925; US; Australia; (-; -); (KC428187; KC428271; KC428364) |
| Corfield 2089; CANB; Australia; (1 KJ661131; -); (-; KJ661183; KJ661213) |
| Corfield 2093; CANB; Australia; (-; -); (-; KJ661182; KJ661212) |
| *Sorghum exstans* Lazarides (2*n* = 10) |
| Hartley 239; US; Australia; (-; 1 KC469352); (KC428193; KC428258; KC428365) |
| Corfield 2039; CANB; Australia; (1 KJ661139; -); (-; KJ661184; KJ661214) |
| Corfield 2120; CANB; Australia; (-; -); (-; KJ661185; KJ661215) |
| *Sorghum interjectum* Lazarides (2*n* = 30) |
| Perry 612; US; Australia; (**1 KC428132** ×1, **2 KC428133** ×1; 1 KC469350); (KC428224; KC428267; KC428353) |
| Cowie 10484; CANB; Australia; (3 KJ661137; -); (KJ661160; KJ661186; KJ661216) |
| Cowie 10513; CANB; Australia; (4 KJ661138; -); (-; KJ661187; KJ661217) |
| *Sorghum intrans* F. Muell. ex Benth. (2n = 10) |
| Celarier A2785-I; US; Australia; (**1 KC428115** ×2; 1 KC469351); (KC428240; KC428313; KC428342) |
| Corfield 2112; CANB; Australia; (-; -); (-; -; KJ661219) |
| *Sorghum plumosum* (R. Br.) P. Beauv. (2*n* = 10/20/30) |
| Cowie 9562; CANB; Australia; (-; -); (KJ661161; KJ661188; KJ661220) |
| Peterson 14419; US; Australia; (-; **1 KJ661143** ×3, **2 KJ661144** ×5, **3 KJ661145** ×1); (KJ661162; KJ661189; KJ661221) |
| *Sorghum stipoideum* (Ewart & Jean White) C.A. Gardner & C.E. Hubb. (2*n* = 10) |
| Fryxell & Craven 4025; US; Australia; (**1 KC428112** ×6, **2 KC428113** ×1, **3 KC428114** ×2; **1 KC469341** ×2); (-; -; -) |
| Liu 242 (PI 562655); IBSC and US; Australia; (**4 KC428122** ×3, **5 KC428127** ×7; **2 KC469336** ×1, **3 KC469337** ×1, **4 KC469338** ×3); (KC428233; KC428280; KC428367) |
| Simon 3968; K; Australia; (-; -); (KJ661163; KJ661190; KJ661222) |
| Subgenus *Chaetosorghum* |
| *Sorghum macrospermum* E.D. Garber (2n = 40) |
| Adams 889; US; Australia; (-; **1 KC469353** ×1, **2 KC469355** ×1, **3 KC469357** ×3); (KC428200; KC428281; KC428359) |
| Corfield 716; CANB; Australia; (-; -); (-; KJ661170; KJ661197) |
| Andrew 374-28; CANB; Australia; (-; -); (-; KJ661171; KJ661198) |
| Andrew 374-29; CANB; Australia; (-; -); (-; KJ661172; KJ661199) |
| Subgenus *Heterosorghum* |
| *Sorghum laxiflorum* F.M. Bailey (2n = 40) |
| Liu 244 (PI 562254); IBSC and US; Sudan; (**1 KC428138** ×2, **4 KC428141** ×1, **5 KC428143** ×2; **1 KC469377** ×2); (-; -; -) |
| Hartley 245; US; Australia; (**2 KC428139** ×1, **3 KC428140** ×1; 2 KC469376); (KC428199; KC428282; KC428339) |
| *Apluda mutica* L. (2n = 20[43]) |
| Liu 252 (PI 215631); IBSC and US; India; (1 KC428182; -); (KC428249; KC428299; KC428381) |
| Liu 253 (PI 271556); IBSC and US; India; (2 KC428183; -); (-; -; -) |
| *Bothriochloa ischaemum* (L.) Keng (2n = 40[43]) |
| Liu 249 (PI 218060); IBSC and US; Pakistan; (1 KC428185; -); (-; -; -)  *Bothriochloa bladhii* (Retz.) S.T. Blake (2n = 40[44])  Liu 250 (PI 208915); IBSC and US; South Africa; (-; **1 KC469416** ×2); (KC428243; KC428307; KC428374) |
| *Chrysopogon serrulatus* Trin. (2n = 20[43])  Liu 255 (PI 219580); IBSC and US; Pakistan; (**1 KC428102** ×2; **1 KC469406** ×4, **2 KC469409** ×1); (KC428250; KC428309; KC428384)  Santos 6311; US; Philippines; (**2 KC428104** ×3; **3 KC469402** ×3); (-; -; -)  *Cleistachne sorghoides* Benth. (2n = 36[2])  Jackson s. n.; US; Malawi; (**A1 KC428169** ×3, **B1 KC428136** ×2; **A1 KC469411** ×1, **B1 KC469412** ×1); (-; -; -)  Reekmans 12070; US; Burundi; (**B2 KC428137** ×3; **A2 KC469410** ×1, **B2 KC469413** ×1); (KC428231; KC428298; KC428370) |
| *Dichanthium annulatum* (Forssk.) Stapf (2*n* = 40[43]) |
| Liu 248 (PI 301974); IBSC and US; India; (-; **1 KC469418** ×2); (KC428245; KC428304; KC428372) |
| Liu 256 (PI 293198); IBSC and US; Thailand; (1 KC428184; **2 KC469420** ×1, **3 KC469421** ×1); (-; -; -)  *Microstegium vimineum* (Trin.) A. Camus (2*n* = 40[45])  Liu 251 (PI 659331); IBSC and US; China; (-; **1 KC469397** ×1, **2 KC469398** ×1, **3 KC469399** ×1, **4 KC469400** ×2); (KC428246; KC428308; KC428371) |
| *Sorghastrum nutans* (L.) Nash (2*n* = 20[46]) |
| Liu 245 (PI 434347); IBSC and US; USA; (1 KC428107; 1 KC469414); (KC428253; KC428302; KC428379) |
| Liu 246 (PI 476279); IBSC and US; USA; (2 KC428108; 2 KC469415); (-; -; -) |

Data listed in order: Accession number (ILRI, International Livestock Research Institute at Addis Ababa, Ethiopia; IS, International Crops Research Institute for the Semi-Arid Tropics at Andhra Pradesh, India; PI, Germplasm Resources Information Network of United States Department of Agriculture at Beltsville, USA); Herbarium (IBSC, South China Botanical Garden Herbarium; CANB, Australian National Herbarium; K, Royal Botanic Gardens, Kew; US, United States National Herbarium); Country of origin; GenBank accession numbers of LCN markers (*Pepc4*; *GBSSI*) followed by sequence ranking number (Prefix “A” or “B” indicates A- or B- type *Pepc4* and *GBSSI* homoeologue for allotetraploid species; interrupted line indicates unavailable sequence) for each species; GenBank accession numbers of plastid genes (*ndhA* intron; *rpl32-trnL*; *rps16* intron) from the sequenced sample in parentheses. Chromosome numbers are based on <http://mobot.mobot.org/W3T/Search/ipcn2.html>. Cloned sequence is labeled in bold and clone number obtained for each sequence is labeled in a multiplication sign and digital, e.g. ×1.
